# Environmental and Molecular Modulation of Motor Individuality in Larval Zebrafish

**DOI:** 10.3389/fnbeh.2021.777778

**Published:** 2021-12-06

**Authors:** John Hageter, Matthew Waalkes, Jacob Starkey, Haylee Copeland, Heather Price, Logan Bays, Casey Showman, Sean Laverty, Sadie A. Bergeron, Eric J. Horstick

**Affiliations:** ^1^Department of Biology, West Virginia University, Morgantown, WV, United States; ^2^Department of Mathematics and Statistics, University of Central Oklahoma, Edmond, OK, United States; ^3^Department of Neuroscience, West Virginia University, Morgantown, WV, United States

**Keywords:** zebrafish, inter-individual variation, individuality, environment, Notch, Gsx, modulation, thermoregulation

## Abstract

Innate behavioral biases such as human handedness are a ubiquitous form of inter-individual variation that are not strictly hardwired into the genome and are influenced by diverse internal and external cues. Yet, genetic and environmental factors modulating behavioral variation remain poorly understood, especially in vertebrates. To identify genetic and environmental factors that influence behavioral variation, we take advantage of larval zebrafish light-search behavior. During light-search, individuals preferentially turn in leftward or rightward loops, in which directional bias is sustained and non-heritable. Our previous work has shown that bias is maintained by a habenula-rostral PT circuit and genes associated with Notch signaling. Here we use a medium-throughput recording strategy and unbiased analysis to show that significant individual to individual variation exists in wildtype larval zebrafish turning preference. We classify stable left, right, and unbiased turning types, with most individuals exhibiting a directional preference. We show unbiased behavior is not due to a loss of photo-responsiveness but reduced persistence in same-direction turning. Raising larvae at elevated temperature selectively reduces the leftward turning type and impacts rostral PT neurons, specifically. Exposure to conspecifics, variable salinity, environmental enrichment, and physical disturbance does not significantly impact inter-individual turning bias. Pharmacological manipulation of Notch signaling disrupts habenula development and turn bias individuality in a dose dependent manner, establishing a direct role of Notch signaling. Last, a mutant allele of a known Notch pathway affecter gene, *gsx2*, disrupts turn bias individuality, implicating that brain regions independent of the previously established habenula-rostral PT likely contribute to inter-individual variation. These results establish that larval zebrafish is a powerful vertebrate model for inter-individual variation with established neural targets showing sensitivity to specific environmental and gene signaling disruptions. Our results provide new insight into how variation is generated in the vertebrate nervous system.

## Introduction

Inter-individual variation, or individuality, is a hallmark of nearly all animal species and contributes to the population’s fitness and ability to adapt when confronted with environmental change (Dingemanse et al., 2004; Klein et al., 2017; Horváth et al., 2020). One form of inter-individual variation is sensory and motor biases. Handedness in humans is a familiar example, expressed as a sustained preference for left- or right-hand use, which the expression of a specific hand preference is only partially explained by genetics, suggesting complex interactions contribute to imposing handed phenotypes (Cuellar-Partida et al., 2020). Indeed, significant complexity underlies human handedness. The preferred hand usage is context-dependent, largely independent of other behavioral biases, and shows variable consistency – observed as consistent or inconsistent hand dominance in a task dependent manner (Watson and Kimura, 1989; Souman et al., 2009; Chu et al., 2012). Handed biases are also a conserved form of individual behavioral variation with species as diverse as hagfish (Miyashita and Palmer, 2014), *Drosophila* (Kain et al., 2012; Buchanan et al., 2015), chicken (Rogers, 1982; Casey and Karpinski, 1999), and various vertebrate paw/foot biases (Bulman-Fleming et al., 1997; Brown and Magat, 2011; Giljov et al., 2013; Schiffner and Srinivasan, 2013; Manns et al., 2021) showing sustained individual motor preferences. Despite the prevalence of handed behaviors, mechanisms instructing these behaviors and the variation observed across individuals are still poorly understood.

Research to date shows that binary handed-like behavioral variation is observed in isogenic *Drosophila* (Kain et al., 2012; Buchanan et al., 2015; Linneweber et al., 2020) and clonal fish (Izvekov et al., 2012; Bierbach et al., 2017). Even more complex behavioral modalities in isogenic mouse strains (Freund et al., 2013, 2015; Hager et al., 2014), *Caenorhabditis elegans* (Stern et al., 2017), *Drosophila* (Linneweber et al., 2020), and clonal crayfish (Vogt et al., 2008) species display stable individual phenotypes with significant inter-individual variation at the population level, suggesting external events contribute to behavioral diversity across individuals. Even in humans, external or stochastic factors are likely important as discordant handedness is frequently observed in monozygotic twins (Jäncke and Steinmetz, 1995). These examples suggest that environmental, chemical, or physical events during development, even at early developmental stages, could produce inter-individual differences. In *Drosophila*, the availability of numerous isogenic strains and the ability to assay large numbers of individuals have been instrumental in elucidating key components generating inter-individual variation (Buchanan et al., 2015). When navigating in their environment, *Drosophila* display a turn bias, where individuals preferentially use same-direction turns, and the magnitude of this bias is modulated by genetic background, activity in the central complex, and exposure to environmental enrichment as well as social experiences (Ayroles et al., 2015; Buchanan et al., 2015; Akhund-Zade et al., 2019; Versace et al., 2020). These findings demonstrate that functional variation in the invertebrate nervous system is maintained by specific neural substrates and further modified by gene and environment interaction. In murine models, exploratory behavior is a thoroughly investigated example of inter-individual variation, where phenotype variation is enhanced by environmental enrichment and correlated changes in hippocampal neurogenesis (Freund et al., 2013; Körholz et al., 2018; Zocher et al., 2020). Despite this well-studied mammalian model and other known handed behaviors that suggest changes in neuron number or activity patterns may regulate inter-individual variation, the mechanisms instructing inter-individual differences remain poorly understood. Therefore, two prevailing questions are what neural substrates generate biases and what mechanisms instruct specific bias types, i.e., left versus right-handed or consistent versus inconsistent handedness.

Zebrafish have emerged as a powerful model for elucidating mechanisms that instruct visceral and neural differences between individuals (Gamse et al., 2003, 2005; Dreosti et al., 2014). Moreover, similar to other teleost species, zebrafish have a visual bias, preferentially using the left eye to assess novelty (Bisazza et al., 1997; De Santi et al., 2001; Sovrano, 2004; Andrew et al., 2009). However, this behavioral bias is primarily fixed in the population and offers little insight into inter-individual variation. Larval zebrafish also perform a light-search behavior that is onset by the loss of visual navigating cues, which drives a period of stereotypic leftward or rightward circling (Horstick et al., 2017), consistent with search patterns observed in other species (Bell et al., 1985; Hills et al., 2004, 2013; Gray et al., 2005). An individual’s leftward or rightward circling direction is persistent over at least multiple days, observed at equal proportions in the population, and is not heritable (Horstick et al., 2020). The features of light-search share many of the hallmark traits observed in well-established invertebrate models of turn bias that have been instrumental for characterizing mechanisms that regulate inter-individual variation (Ayroles et al., 2015; Buchanan et al., 2015; Akhund-Zade et al., 2019). Moreover, our work has shown that neurons in the habenula and rostral posterior tuberculum (PT) are essential for maintaining zebrafish turn bias (Horstick et al., 2020). Therefore, larval zebrafish is a potentially powerful vertebrate model to determine how inter-individual variation is imposed in the vertebrate brain. What remains lacking is a rigorous analysis of turn bias variation in the population and the identification of external and internal factors modulating inter-individual turn bias differences.

Here, we capitalize on the larval zebrafish turning bias to characterize environmental factors and signaling pathways that modulate inter-individual variation. Previous work identified a persistent left/right turn bias maintained by a habenula-rostral PT circuit and Notch associated signaling pathways (Horstick et al., 2020). However, locomotor features or factors instructing turn direction phenotypes was unexplored. Here we develop a multiplex recording pipeline and a new metric, bias ratio, which permit turn bias recording in a medium-throughput manner and rigorous unbiased analysis of inter-individual variation. Previous work used metrics that weighted behavior on a single trial to categorize turning type (Horstick et al., 2020), which these metrics are potent indicators of bias, yet can easily compound error over serial testing that is typically required to study probabilistic behavior like turning bias. Using our new testing pipeline, we first characterized turning types, finding previously described left and right turning types and a previously undescribed unbiased turning type in a wildtype strain. We further establish that turning types are distinguishable by unique path trajectory features. Second, we determined that temperature selectively impacts inter-individual variation and rostral PT neurons, establishing a tentative mechanism for temperature dependent regulation of inter-individual variation. Last, we investigate molecular pathways, demonstrating a direct role for Notch signaling using pharmacological inhibition. We establish levels of Notch inhibition that disrupts habenula development and bias, yet well-established Notch mechanisms such as neuronal proliferation or morphological development are unaffected. By testing a mutant associated with Notch signaling, gsx2, we implicate that brain regions beyond that previously described circuit could be important for developing variation in a vertebrate. This work develops zebrafish search behavior as a model for inter-individual variation and reveals how environmental and molecular cues impact specific neural substrates to generate distinct behavior types in a vertebrate.

## Materials and Methods

### Animal Husbandry

All experiments were approved by the West Virginia University Institutional Animal Care and Use Committee. Zebrafish (*Danio rerio*) Tübingen long-fin (TL) wildtype strain was used in all experiments and used as the genetic background to maintain transgenic and mutant lines. Experiments were conducted during the first 7 days post fertilization (dpf), which is before sex determination. Larval rearing conditions were 28°C, 14/10 h light-dark cycle, in E3h media (5 mM NaCl, 0.17 mM KCl, 0.33 mM CaCl_2_, 0.33 mM MgSO_4_, and 1 mM HEPES, pH 7.3), and at a stocking density of 40 embryos per 30 mL E3h, unless stated otherwise. *Social environment*: To test the effect of social interaction, we raised larvae under two different social conditions: 20 larvae in a 6 cm petri dish or a single larva per 6cm dish. Social or isolation rearing started at 5–8 h post fertilization (hpf) and continued until testing at 6 dpf. *Temperature*: To test the impact of temperature on the development of turn bias, larvae were raised 1–4 dpf at either 24, 28, or 32°C. At 4 dpf, all groups were moved to 28°C until testing at 6 dpf. To determine if a specific development period was sensitive to elevated temperature, separate groups of larvae were raised at 32°C from either 31–55 hpf or 55–79 hpf, after which they were returned to standard rearing temperature and tested at 6 dpf. *Salinity*: The impact of increased salinity was tested over 4 salt concentrations (1, 2, and 5 ppt – parts per thousand) and standard E3h (∼0.5 ppt) as a control. Larvae were reared in variable salinities from 1 to 4 dpf, and behavior tested at 6 dpf. An elevated salinity stock of E3h was made by adding 9.5g NaCl (Sigma) to standard E3h, creating a 10 ppt stock, which was diluted for working concentrations with standard E3h media. *Environmental enrichment:* Enriched environments were created by adhering mixed size and color (predominately red, blue, gray, and white colors) LEGO^®^ blocks onto the bottom of a 10 cm petri dish. Previously, LEGO^®^ blocks have been used to stimulate novel object recognition and interaction in larval zebrafish (Bruzzone et al., 2020). In addition, 5–8 plastic aquarium leaves were included to float on the surface. Last, dishes were positioned on platforms with mixed white and black shape substrates. A total of four enriched environments were created with variable LEGO^®^ colors and sized blocks, and larvae were rotated daily between enriched environments. As controls, larvae were raised in plain 10 cm dishes placed on either a solid white substrate. For experiments, larvae were maintained in enriched or control dishes from 1 dpf until behavior testing. *Shaking*: We tested the impact of environmental instability on motor bias by continuously shaking larvae from 1 to 4 dpf. At 1 dpf, embryos were placed in a 75 cm^2^ cell culture flask (Sigma) with approximately 80 mL E3h. Flasks were propped at 30 degrees on a Stovall Belly Dancer orbital rotator, set to 70 rpm. At 4 dpf, larvae were removed from culture dishes and raised under standard conditions prior to testing at 6–7 dpf.

Transgenic lines used were enhancer trap *Tg(y279-Gal4)* (Marquart et al., 2015) and *Tg(UAS:Kaede)s1999t* (Davison et al., 2007). Mutant line used was *gsx2*^Δ13*a*^ (Coltogirone et al., 2021).

### Behavior Tracking and Analysis

Behavioral experiments were performed on 6–7 dpf larvae, except as noted. All experiments were recorded using infrared illumination (940 nm, CMVision Supplies), a μEye IDS1545LM-M CMOS camera (1st Vision) fitted with a 12 mm lens, and a long-pass 780 nm filter (Thorlabs, MVL12WA and FGL780, respectively). Visible illumination was provided by a white light LED (Thorlabs) positioned above the larvae, adjusted to 40–50 μW/cm^2^ (International Light Technologies, ILT2400 Radiometer with SED033 detector). Testing conditions were maintained between 26 and 28°C for all behavioral recording, and all larvae adapted to the recording room conditions for 20 min before recording under matched illumination to recording rigs. Custom DAQtimer software was used to control lighting, camera recording parameters, and real-time tracking as previously described (Yokogawa et al., 2012; Horstick et al., 2017). The camera field of view was set to record four 10 cm dishes simultaneously with one larva per dish for multiplex recordings. A total of four recording rigs were used. Path trajectories of individual larvae are recorded over 30-s recording intervals at 10 fps and analyzed using five measures: net turn angle (NTA), total turning angle (TTA), match index (MI), bias ratio (BR), and performance index (PI) (see Table 1 for metric reference). A minimum of 100 points were required to be included in the analysis. NTA is the summation of leftward and rightward angular displacement (−leftward, +rightward) over the recording interval, whereas TTA is the sum of absolute values of all angular displacement. MI measures the proportion of events in a series going in the same direction. Leftward and rightward trials are scored as 0 or 1, and MI is the percent of events matching the direction of the first trial in a testing series. For example, a MI = 1 is all events are in the same direction as the first trial, whereas 0.33 is a third of the events matching the first trial. For MI analysis, individuals missing the first dark trial were excluded from analysis. BR is a proportion of directional turning compared to total turning, calculated by dividing NTA by TTA, e.g., −1 represents that all directional movement in a single trial occurred in a leftward direction, while −0.5 indicates that 50% of all turning was in a net leftward direction (e.g., −200 degrees NTA out of 400 TTA). PI was calculated by averaging binary bias ratios, with leftward trials scored as 0 and rightward 1. Where noted on figures, bias ratios were weighted by the proportion of larvae within a PI group in order to demonstrate changes in the number of larvae within a performance group. In all analyses that required a PI for categorizing larvae, all individuals that had missing trials were excluded. This criteria was necessary to ensure rigorous categorization. For *gsx2* experiments, larvae were housed individually following behavior testing for *post hoc* genotyping. Genotyping was performed as previously described (Coltogirone et al., 2021). In brief, genotypes were confirmed using PCR spanning the deletion: *gsx2* (primers: 5′TGCGTATCCTCACACATCCA, 5′TGTCCAGGGTGCGCTAAC; 134 bp wildtype, 121 bp mutant, and 134/121 bp heterozygous). Previous reports describe that gsx2 mutants have reduced swim bladder inflation (Coltogirone et al., 2021), which was minimized by raising larvae in shallow water dishes. Only larvae with normal swim bladder inflation and balance were used for experiments.

**TABLE 1:** Reference for metrics and assays.

Behavior metrics	Name	Measure
NTA	Net turn angle	Net sum of leftward and rightward angular movement
TTA	Total turn angle	Absolute sum of all angular movement
BR	Bias ratio	NTA/TTA ratio. Proportion of directional movement
MI	Match index	Proportion of trials matching direction of first trial
PI	Performance index	Average of binarized turn directions (0 = left; 1 = right)

**Behavior assays**	**Name**	**Measure**

4×	NA	Paired 30 s Light ON and OFF recordings, repeated 4×
8×	NA	Paired 30 s Light ON and OFF recordings, repeated 8×
q4×	Quad 4×	Four repeated ‘4×’ recordings separated by 10 min

The 4× recording assay was performed by recording larval path trajectories over four recording intervals, each composed of 30 s baseline recordings, immediately followed by 30 s recording following the loss of visible illumination. Each recording interval was separated by 3 min of baseline illumination. The 8× recording was performed in a similar format, including four additional light ON/OFF recording intervals performed in series. The quad 4× (q4×) assay is identical to the 4×, except that the 4× recording interval is repeated four times, separated by 10 min baseline illumination (see Table 1 for assay reference). A 4× recording strategy was used to test the developmental onset of turn bias. Individual larvae were first tested at 3 dpf, and were separately raised in 6-well plates and retested daily through 6 dpf. For analysis, larvae were grouped as left or right biased based on BR (average BR+, right bias; −, left bias) at 6 dpf when turn bias is well-established (Horstick et al., 2020). To ensure rigorous categorization, larvae with ambiguous responses at 6 dpf (BR between −0.1 and 0.1) were removed.

### Pharmacology

Notch signaling was inhibited using the Υ-secretase inhibitor LY411575 (Sigma, SML050). A 10 mM stock of LY411575 was prepared in DMSO and diluted to working concentrations with a final volume of 0.08–0.1% DMSO for all trials. To test Notch inhibition on turn bias, mid-gastrulation (6–8 hpf) groups of embryos were treated with 0.05, 0.1, 0.15, 0.2, 0.25, 1 or 10 μM LY411575 until 4 dpf; the drug was replaced daily. At 4 dpf, LY411575 was removed and larvae placed in fresh E3h until behavior testing at 6 dpf. Phenotypic categorization was performed at 3 dpf. Individuals were scored as normal (visually no abnormal tail curvature, edema, reduced/decreased swim bladder size, necrosis, or overt abnormal swimming), mild (abnormal touch responsiveness), moderate (tail curvature), or severe (gross developmental defects, necrosis). Only normal larvae were used for behavioral testing. Vehicle controls were 0.08–0.1% DMSO treated.

### Labeling and Imaging

#### Immunohistochemistry

To assay neuronal proliferation, we labeled with anti-HuC/D (Elav protein) (Invitrogen A21271). Control (0.08% DMSO) and LY411575 groups (100 nM and 8 μM) were prepared as described above. At 24 hpf, embryos were fixed overnight using 4% paraformaldehyde in 1× PBS at 4°C. Washes were performed with 1× PBS containing 0.1% TritonX-100. We used primary antibody mouse anti-HuC/D (1:500, Invitrogen, 16A11). Secondary detection was performed with goat anti-mouse IgG1 Alexa 488 (1:500, Invitrogen, A32723). To analyze images, signal intensity of a 56 μm × 6 μm (W × H) region spanning a lateral to midline hemi-section of the anterior spinal cord was recorded using ImageJ. Three sections were measured per larva, averaged and standardized for comparison between groups.

#### Fluorescent *in situ* Hybridization

To determine the levels of Notch signaling we examined transcript levels of her12 (Jacobs and Huang, 2019). Hybridization chain reaction (Molecular Instruments) probes and labeling technology was used to detect her12 transcripts. Her12 mRNA sequence (NM_205619) was provided to Molecular Instruments to design a custom gene-specific her12 probe detection set. LY411575 and control larvae were treated as described above. At 30 hpf, larvae were fixed overnight using 4% paraformaldehyde in 1× PBS at 4°C. Fixed larvae were washed in 1× PBS containing 0.1% Tween20 and labeled following Molecular Instruments HCR RNA-Fish protocol for whole-mount zebrafish embryos (Schwarzkopf et al., 2021). All images were collected using the same parameters. For analysis, the percent area of her12 expression was quantified within the spinal cord using ImageJ.

#### Neuron Temperature Sensitivity

Rostral PT and habenula image stacks were captured and neurons counted in max projections using ImageJ. All imaging was performed using larvae from *Tg(y279-Gal4)/Tg(UAS:kaede*) carrier in-crosses. At 1 dpf, larvae were screened for Kaede and reared at elevated temperatures as described above. Larvae were moved to standard raising conditions at 4 dpf, and live-imaged at 6 dpf. Larvae were anesthetized using MS-222 (Sigma) and mounted in 2% low melting temp agar. To determine if a specific developmental time period was crucial, larvae were similarly prepared and analyzed, yet only raised at elevated temperature during either 31–55 hpf or 55–79 hpf intervals. Controls were raised at standard rearing temperatures.

#### Neuron Sensitivity to Notch Inhibition

Using *Tg(y279-Gal4)/Tg(UAS:kaede*) carrier in-crosses we performed LY411575 as described above, except treatments ended at 3 dpf when both the habenula and PT could be observed, while attempting to minimize severe morphological phenotypes and death at higher concentrations. We treated embryos at concentrations of 0.1 and 1 μM with a vehicle control. All treatments had a final DMSO concentration of 0.01%. Imaging the habenula and PT was performed as above and neuron counts performed using max projections in ImageJ. Counts were only performed on groups were habenula and PT neurons could be reliable identified. For counting neuron numbers, the larger habenula was classified as the ‘left’ habenula regardless of hemisphere. Habenula were classified as symmetric if the left to right neuron ratio was less than 2.

#### Imaging

All imaging was performed on an Olympus Fluoview FV1000. For live imaging, larvae were anesthetized in a low dose of MS222 (Sigma) and embedded in 2% low melting temp agar. Fixed samples were transferred into 70% glycerol/30% 1× PBS and slide-mounted for imaging.

### Statistical Analysis

Analysis was performed in R R Core Team (2020). R: A language and environment for statistical computing, 2020), R ggplot2 package (Wickham, 2016) (R Core Team) and Prism (GraphPad). All statistical comparisons were two-sided, unless noted otherwise. Standard error of the mean (±SEM) was used for all experiments, except MAD analysis which display 95% confidence intervals. Cohen D was calculated in R using package effsize. For all experiments, data was collected from a minimum of three independent clutches. Normality was tested using the Shapiro–Wilks test. Normally distributed data was compared using either 1 or 2-way *t*-tests. Non-normal data was analyzed using a Mann–Whitney *U* test or Wilcoxon signed-rank test for 2 or 1-way tests, respectively. To perform multiple comparisons, ANOVAs were performed in GraphPad and multiple comparisons adjusted using a Bonferroni correction. Boxplots show median and quartiles with outliers identified beyond 2.7 standard deviations from the mean.

Permutation and bootstrapping was performed using “sample” R function without and with replacement, respectively. For permutation experiments, bias ratios values were randomized across all individuals in a dataset. Randomization was performed only within the same trial, e.g., reshuffling of bias ratios within the first light off trial. Permutations were simulated 1,000 times and average bias ratios and MAD values calculated using custom R code, and used to plot permutated probability density curves and MAD values. Probability density plots and area under the curve measurements were performed using custom R code. For area under the curve analysis, ±0.3 tails were chosen for comparison, which are approximately two standard deviations from the population average. To generate error bars for MAD analysis, average bias ratios were bootstrapped (1,000 bootstrap replicates) with resampling. For each resampled dataset a MAD was calculated and MAD values across all resampled datasets used to calculate a 95% confidence interval applied as an error bar. A 1-way comparison was used to calculate significance for all simulated dataset comparisons. To generate a *p*-value, the number of resampled dataset MAD values were totaled that fall within or exceed the 95% confidence interval of the comparison group, and this total was divided by 1,000 to produce a final *p*-value. This represents the fraction of simulated experimental groups that fall within a range that supports a null hypothesis of no difference between groups. For example, 600 bootstrapped datasets from a simulated control that fall within or exceed the confidence interval of an experimental group yields *p* = 0.60, implicating that 60% of simulated datasets do not support the statistical difference between compared groups. Direction of comparison is noted in the legend for each dataset.

## Results

### Turning Behavior During Light Search Shows High Inter-Individual Variation

**FIGURE 1:**
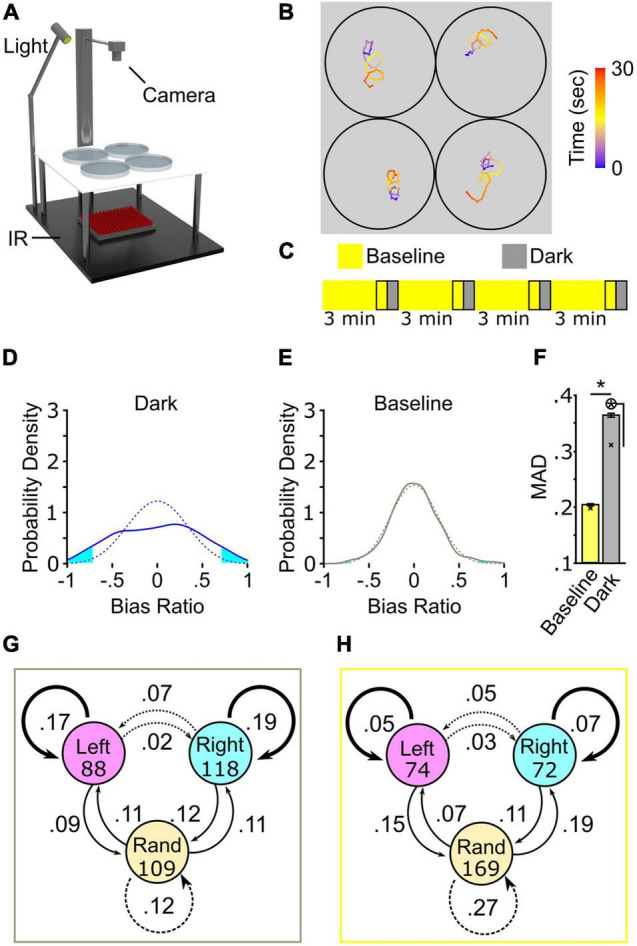
Turning behavior following the loss of light exhibits high inter-individual variation. **(A)** Schematic of multiplex recording rig. **(B)** Illustrative path trajectories following loss of light from multiplex recording. Color scale is time (seconds). **(C)** Diagram of 4× recording. Outlined regions denote recording intervals. **(D–F)** 4× recordings strategy to show high inter-individual variation in turn bias. **(D)** Average bias ratio probability density curve for dark responses (solid blue line, *N* = 374) and paired curve following reshuffling (dotted blue line, average of 1,000 resampled datasets). **(E)** Same as **(D)** except the paired baseline responses (solid gray line, *N* = 374) and random permutation density curve (dotted gray line). Cyan fill shows the area in each tail corresponding to the probability of observing a result more extreme or equal to ±0.3 average bias ratio. **(F)** MAD for baseline (yellow), and dark (gray) responses. *N* = 374. Error bars are 95% confidence intervals generated by bootstrap resampling. Asterisk in circle, *p* < 0.05 to MAD permutation value shown with an X. **(G)** Dark responses (gray outline) and baseline **(H)** (yellow outline) from 4× recording showing frequency of direction change between first (circles) and second (arrows) pairs of responses. Initial *N* shown in circles. Bold, solid, and dotted arrows delineate responses that produce perfectly matched bias, partial bias, and unbiased responses, respectively.

We developed a multiplexed strategy to record path trajectories to assess inter-individual variation during larval zebrafish light search behavior (Figure 1A). Previously, the stereotypic turning was described using a large recording arena (14,400 mm^2^) to record single larva (Horstick et al., 2017). Larvae are recorded in 100 mm diameter dishes (7,854 mm^2^) for our multiplexed strategy, and robust circling is observed following light extinction (Figure 1B). To characterize individual motor biases, we initially recorded larval path trajectories over a series of four intervals of paired 30-s baseline illumination and 30 s following the loss of illumination, with each of these recording pairs separated by 3 min of illumination to restore baseline behavior (Horstick et al., 2020), which we refer to as 4× recording (Figure 1C). This recording yields four paired light on and off events per individual. We recorded responses from 374 individuals, representing 1,496 paired baseline and dark responses. The presence of motor bias was previously described using a match index (MI) – the percent of turning trials in which turning direction was the same as the first dark trial (Horstick et al., 2020). Here we confirm previous findings showing a significant MI increase following the loss of illumination (Wilcoxon matched-pairs test, *p* < 0.001), showing the number of individuals recorded can be upscaled via multiplexing (Supplementary Figure 1A). Overall, our current approach for multiplexed recording recapitulates previous findings. These data show that our multiplexed strategy provides medium-throughput recording, allowing a rigorous analysis of larval zebrafish inter-individual variation.

We calculated a bias ratio by dividing net turning angle (NTA) by total turning angle (TTA – absolute sum of all angular displacement) for each baseline, and dark trial recorded to examine the spectrum of wildtype larvae inter-individual variation during search behavior (Supplementary Figure 1B). This metric provides the proportion of same-direction turning within a continuous numerical range bounded by −1 and 1, representing all directional movement in a leftward or rightward direction, respectively. The average bias ratio across the entire population during baseline illumination and light-search did not significantly vary from zero showing no population bias [one-sample *t*-test against 0, baseline: *t(373)* = 0.007842, *p* = 0.9937; dark: *t(373)* = 0.1696, *p* = 0.89] (Supplementary Figure 1C). Despite similar population-level bias ratios between baseline and dark, significant variation is observed in the dark that is not observed during baseline (Figures 1D–F). Using a probability density curve, where the area under the curve represents the proportion of individuals in the population, we find that during dark turning, 12.38% of the population displayed a robust sustained turning bias over 4 trials (bias ratio <−0.7 = 6.41%, left bias; >0.7 = 5.97%, right bias) (Figure 1D). Conversely, 1.72% of baseline events displayed sustained directional turning (Figure 1E). The distribution of bias ratios shows that, following light extinction, a significantly greater number of individuals utilize sustained same-direction turning [χ^2^(1) = 51.02, *p* < 0.0001]. To determine whether these distributions were the product of chance, we simulated ‘randomized’ baseline and dark datasets by resampling bias ratios (1,000 resamples) within each trial (Figures D,E, dotted line). Following randomizing, 2.35% of the simulated dark responses maintained strong directional turning, similar to that observed during baseline. A previous study used mean absolute deviation (MAD) as a metric to quantify variation in a population; a higher MAD represents increased variation across individuals in the population (Buchanan et al., 2015). Here, MAD was calculated for baseline, dark, and simulated datasets. As MAD was generated from the whole population, average bias ratios were bootstrapped (1,000 boots) to generate 95% confidence intervals for statistical comparison. MAD is 44.10% (*p* < 0.001) and 15.79% (*p* < 0.001) reduced in baseline or in randomized dark groups compared to light-search dark responses, respectively (Figure 1F), whereas no difference was observed between baseline MAD and randomized baseline responses (Figure 1F, yellow bar). These findings show that turn bias during light search behavior shows significant variation beyond what is expected by chance or while larvae navigate in an illuminated environment.

Our analysis, along with findings from previous reports, illustrates robust left and right turners, or turning types, within the population. However, the distribution of bias ratios from 4× recordings shows that over 14% of the population exhibits an average bias ratio consistent with no sustained turn direction (−0.1 < BR < 0.1) (see Figure 1D). These individuals could represent either a stable unbiased population or endogenous behavioral fluctuation. To evaluate whether unbiased individuals are a sustained turning type in the population, in addition to left/right biased turners, we created a performance index (PI) by transforming all individual trials to either 0 or 1 for overall leftward or rightward preference per trial, respectively. From these binary values, we created a transition index for the first and second set of responses from the 4× dataset, i.e., left (LL = 0), right (RR = 1), or random (LR; RL = 0.5) responses that can be compared between the first and last response pairs. Using the transition pair PI, we assessed the frequency of turn direction change or conservation (Figures 1G,H). During dark trials, 36% of all transitions showed sustained turn direction (left = 17%, right = 19%; average PI = 0 or 1), whereas during baseline illumination 12% of larvae sustained turn direction [χ^2^(1) = 54.545, *p* < 0.0001]. Conversely, 21 and 35% of transitions yielded sustained random behavior between dark and baseline recording conditions, respectively (for example, LR to RL or RR to LL; average PI = 0.5) [χ^2^(1) = 8.615, *p* = 0.0033] (Figures 1G,H). Interestingly, during light-search initially random response pairs transitioned to directional (RR or LL) responses 22% of the time, yielding partial turn bias (average PI 0.75 or 0.25).

To confirm our observations persisted over longer timescales, we ran an additional 8× experiment, testing 189 larvae as before, with four additional light ON/OFF intervals in series. From this extended testing condition, we observed conserved trends demonstrating significant inter-individual variation in turning bias during light-search, yet not during baseline illumination (Supplementary Figures 1D–F). Neither 4× or 8× recording showed a change in TTA over time, establishing overall behavior is not disrupted by our assays (Supplementary Figures 1G,H). As 4× and 8× experiments were broadly consistent, we focused on the 4× recording strategy for ongoing investigations. Our data show that wildtype larvae exhibit significant inter-individual variation in turn bias during light-search, greater than that expected by chance, with a subset of individuals potentially exhibiting a previously unexplored unbiased turning type.

### Multiple Stable Turning Types Exist With Distinct Locomotor Features

**FIGURE 2:**
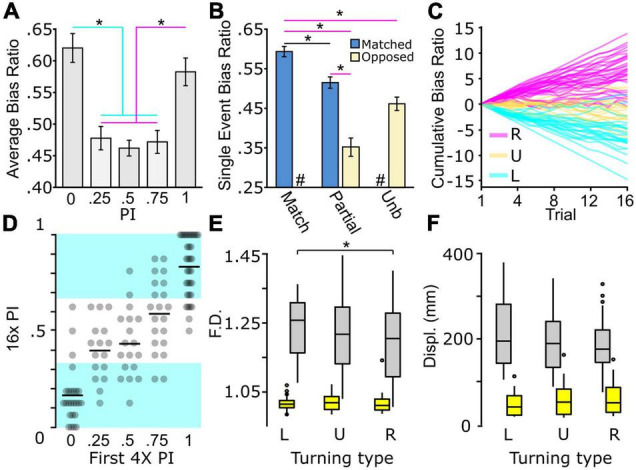
Biased and unbiased motor types present during light-search. **(A)** Absolute bias ratio from 4× recording per PI (PI 0, *N* = 66; PI 0.25, *N* = 74; PI 0.5, *N* = 75; PI 0.75, *N* = 75; PI 1, *N* = 67). **(B)** Absolute bias ratio magnitude for single 30 s recordings occurring in a direction matching (blue) overall average PI direction or occurring in the opposed (beige) direction shown for perfect bias (0,1 PI, *N* = 517), partially biased (0.25, 0.75 PI, *N* = 596), and unbiased (0.5 PI, *N* = 301) populations. **(C)** Cumulative summation of bias ratio over the q4× from strong right (magenta, *N* = 34), strong left (cyan, *N* = 34), and unbiased (yellow, N = 18) individuals. Individuals were selected based on the first 4× average PI (strong right PI = 1, strong left PI = 0, unbiased PI = 0.5). **(D)** Average q4× PI based on initial 4× PI (PI = 0, *N* = 66; PI = 0.25, *N* = 74; PI = 0.5, *N* = 75; PI = 0.75, *N* = 75; PI = 1, *N* = 67). Black bars represent sample mean. Left, right, and unbiased performance categorized within equal thirds of the PI scale. Cyan background highlights average PI with left or right turning type behavior. **(E,F)** Characterization of path trajectory features during baseline (yellow boxes) and dark (gray boxes) responses from individuals tested in the q4× assay. Turning type determined by 16× average PI distribution in **(D)**. **(E)** Fractal dimension and **(F)** Displacement (L, left, *N* = 36; U, unbiased, *N* = 40; R, right, *N* = 38). Asterisk *p* < 0.05.

Characterizing changes in locomotor parameters in zebrafish has been a powerful strategy to develop etiological and mechanistic models (Burgess and Granato, 2007; Horstick et al., 2013; Chen and Engert, 2014; Dunn et al., 2016). Therefore, we next aimed to establish what underlying locomotor changes account for unbiased and biased motor types. We hypothesized that three possible modes could generate unbiased behavior: (1) normal turning with high rates of direction switching across trials, (2) reduced same-direction turning within single trials, or (3) weak photo-responsiveness and, therefore, low total turning. To differentiate between these hypotheses, we categorized all larvae based on average PI across all four trials, generating five categories. Across PI groups, we compared the absolute average bias ratio to determine if the magnitude of directional turning changed based on PI. During light search the average bias ratio magnitude significantly changed based on PI [1-way ANOVA *F(4,352)* = *10.43*, *p* < 0.0001], where partial and unbiased PI groups showed less overall directional turning (Figure 2A). No difference was observed between strong left and right biased turners [PI = 0, 0.603 ± 0.022; PI = 1, 0.58 ± 0.021: *t(352)* = 0.7811 adjusted *p* > 0.9999]. Consistent with earlier observations, no differences were observed across PI groups during baseline [1-way ANOVA *F(4,352)* = *2.087*, *p* = 0.082], consistent with an absence of turn individuality (Supplementary Figure 2A). Moreover, there was no significant change in TTA during dark turning [1-way ANOVA *F(4,352)* = *1.263*, *p* = 0.28] across all PI groups (Supplementary Figure 2B). As all PI groups exhibited normal levels of total turning, this ruled out variable photo responsiveness as the basis of different turning types. Unexpectedly, partially biased populations (0.25, 0.75 PI) showed a similar average bias ratio as unbiased larvae (Figure 2A). To explain this observation, we reasoned that bias ratio magnitude might vary depending on whether an individual trial matches or opposes the overall larva turning type. For example, for 0.25 PI larvae, leftward matched direction bias ratios compared to rightward opposed direction trials. We analyzed all individual trials between all performance groups to explore this idea, sorting trials into matched or opposing based on the average PI for each individual. Perfect performance trials (0,1) were categorized as all matched, whereas unbiased trials (0.5) as all unmatched. Left and right direction bias ratios did not vary in these groups; therefore, we combined these groups to simplify comparison (Supplementary Figure 2C). A significant effect was observed across groups [1-way ANOVA *F(3,1408)* = *27.93*, *p* < 0.0001], with trials opposed to overall PI direction showing lower overall bias ratio strength (Figure 2B, magenta lines). These data suggest that the basis of unbiased motor types is due to a lower bias ratio or less persistent same-direction turning, yet not a loss of overall turning. Interestingly, we noted that matched bias ratios were reduced in partially matched trials compared to events in the fully matched group [match 0.594 ± 0.013: partial match 0.514 ± 0.015: *t(1408)* = 4.046 adjusted *p* = 0.003] (black line), implicating that the underlying differences between biased and unbiased larvae may be graded.

In order to confirm rigorously the three motor types, we performed a quad 4× assay (q4×), using the standard 4× assay repeated four times, with each recording sequence separated by 10 min of baseline illumination (Supplementary Figure 2D). We recorded 114 larvae, and consistent with our previous measures, individuals showed significant inter-individual turn bias variation during light-search (±0.3 probability density tails: 7.34% dark; 0.00037%, randomized dark), and sustained left, right, or unbiased locomotor preferences (Supplementary Figure 2E). The cumulative summation of bias ratios provided a qualitative measure of turn performance over time (Figure 2C). From this analysis, we noted that some larvae initially categorized as strong or unbiased turners, seemingly switched over time. Therefore, we next aimed to utilize the q4× analysis to quantify bias determination accuracy by comparing the first 4× PI to overall q4× performance. We equally divided the 0 to 1 PI scale for classifying left, right, or unbiased behavior (left ≤ 0.33; unbiased 0.33 < 0.66; right ≥ 0.66) (Figure 2D). Of the larvae that show an initial strong or partial bias during the first 4× interval, 2/96 (2.08%) reverse bias direction during the q4× assay, and 27/96 (28.13%) of these individuals ultimately switch to an unbiased response after serial q4× testing. However, switching is primarily observed in larvae showing an initially partial bias, as the larvae that displayed an initially strong bias (0, 1 PI) in the q4× assay, 50/59 (84.75%) maintained a left or rightward turning type. Interestingly, at the population level, the 9/114 (7.89%) of unbiased individuals initially categorized with a strong bias was comparable to that expected by random chance, i.e., the same 6.25% likelihood of flipping 4 heads with a coin [χ^2^(1) = 0.609, *p* = 0.435]. As expected, classifying unbiased larvae was less accurate, yet a single 4× trial accurately represented 10/18 (55.56%) of individuals. Altogether, the q4× testing strategy confirms our earlier findings and demonstrates the veracity of our recording strategies to detect specific turning types.

As the q4× assay allowed for a rigorous confirmation of turning type, we next wanted to determine whether left, right, or unbiased turning types exhibited unique path trajectory characteristics. A PI was calculated from all 16 trials in the q4× assay for each individual and categorized as left, unbiased, or right type. For each turning type, we examined fractal dimension (F.D.) and displacement (displ) as measures of local search behavior (Tremblay et al., 2007; Horstick et al., 2017). Comparison across all three motor types yielded no differences in the tested motor parameters [main effect due to turn type 2-way ANOVA displ: *F(2,222)* = *0.42*, *p* = 0.66; F.D: *F(2,222)* = *2.12*, *p* = 0.12], yet the expected changes in behavior following light extinction were observed [main effect due to illumination 2-way ANOVA displ: *F(1,222)* = *604*, *p* < 0.0001; F.D: *F(1,222)* = *643*, *p* < 0.0001] (Figures 2E,F). Interestingly, upon closer inspection, we did notice a small yet significant change in F.D. between left and right turning groups during dark trials [left 1.240 ± 0.012; right 1.200 ± 0.014: *t(222)* = *2.974*, adjusted *p* = 0.0489, effect size *d* = 0.63]. This effect was specific, and not observed during baseline [left F.D. 1.021 ± 0.003; right F.D. 1.021 ± 0.004: *t(222)* = *0.059*, adjusted *p* > 0.9999] or for displacement. These results show that the difference of left and right turning type also generate mild changes to search pattern behavior, yet not motor trajectories during baseline movement.

### Development of Inter-Individual Variation Is Sensitive to Specific Environmental Factors

**FIGURE 3:**
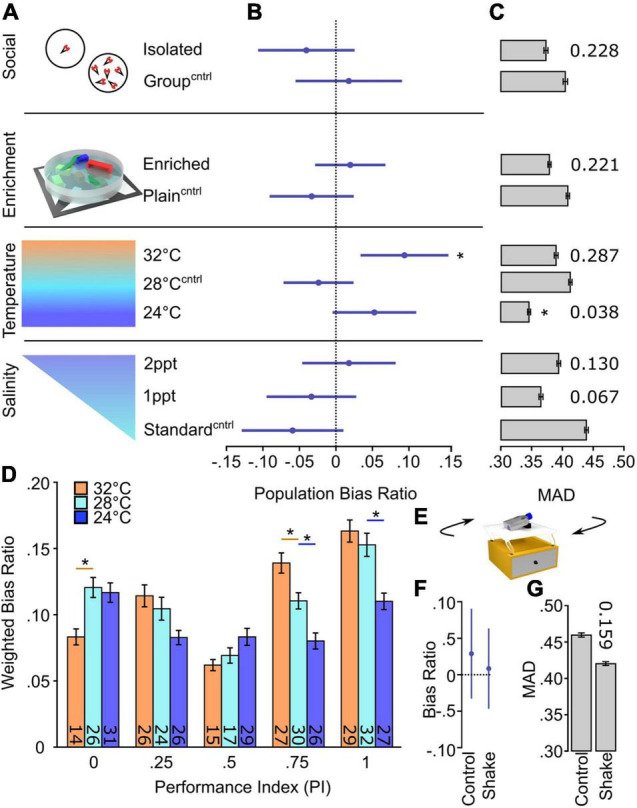
Temperature selectively changes inter-individual variation in turn bias. **(A)** Schematic of environmental manipulations (Social Isolated *N* = 87, Social Group *N* = 87; Enriched *N* = 172, Plain *N* = 134; Temp. 32°C *N* = 112, Temp. 28°C *N* = 136, Temp. 24°C *N* = 144; Standard salinity *N* = 102, 1 ppt *N* = 107, 2 ppt *N* = 107). Each condition has an independent control denoted by superscript Cntrl. **(B)** Average bias ratios across the entire tested population per condition. Statistical comparison performed to 0, denoting no population level bias. **(C)** MAD for dark responses, error bars are 95% confidence levels generated from 1,000 bootstraps. Individual *p*-values shown above bars, calculated by comparing experimental groups to controls. **(D)** Average bias ratio per PI weighted by the percent of individuals. *N*’s indicated within bars. **(E)** Representative diagram of the setup used to shake larvae during early development. Average population bias ratio **(F)** and MAD **(G)** between shake experiments. Number above bar represents *p*-value compared to the control group. Asterisk *p* < 0.05.

Many instances of motor and behavioral biases show limited heritability (Collins, 1969; Buchanan et al., 2015; Linneweber et al., 2020). This observation suggests that inter-individual variation is, at least in part, modulated through individual experience with environmental factors. Indeed, previous studies show that social experience and environmental enrichment modify inter-individual variation of specific behaviors (Freund et al., 2015; Akhund-Zade et al., 2019; Versace et al., 2020; Zocher et al., 2020). As larval zebrafish turning bias is not heritable (Horstick et al., 2020), we reasoned that the environment might contribute to overall inter-individual variation or the generation of specific turning types. To test this hypothesis, we first established that turn bias appears at 4 dpf (Supplementary Figures 3A,B). Therefore, larvae were exposed to changes in the environment from 1 through either 4 or 7 dpf, dependent on the tested factor. The four parameters we screened were social experience, environmental enrichment, temperature, and salinity (Figure 3A). Social interaction and environmental enrichment were selected because each has been shown to modulate inter-individual variation (Akhund-Zade et al., 2019; Versace et al., 2020). For social interaction, larvae are raised in isolation or groups. For enrichment, we generated two environments: (1) an enriched environment where a petri dish was fitted with internal surfaces, diverse color, hiding spots, water surface cover, and dynamic substrate pattern, and (2) an empty petri dish with a uniform white bottom as a control. In addition, we also tested the impact of etiologically relevant temperature (24 or 32°C) and salinity [0.5–5 parts per thousand (ppt)] variations during early development compared to standard rearing parameters (Engeszer et al., 2007; Sundin et al., 2019). Thus, our parameters test factors that generated inter-individual variation in other models and abiotic environmental fluctuations that larvae could encounter in a native habitat.

To determine if any of the tested parameters altered turning type development or magnitude of inter-individual variation, we looked at the average population bias ratio and MAD, respectively (Figures 3B,C) (Supplementary Figure 3C). Interestingly, the elevated temperature during early development caused a significant population shift from random [high temp 0.094 ± 0.044: one-sample *t*-test against 0, *t(112)* = *2.157*, *p* = 0.033], implicating a population-level rightward bias, whereas no significant changes were observed in other temperature conditions or any other tested environmental parameter (Figure 3B). Conversely, the magnitude of turn bias variation during light-search was only reduced by low-temperature rearing, yet unaffected by other testing conditions (Figure 3C). To confirm the observed temperature-dependent changes, we examined the bias ratio per PI, weighted for the number of individuals per PI group. We observed that temperature imposed a significant effect on turn bias persistence [main effect of temperature 2-way ANOVA *F(2,364)* = *9.275*, *p* = 0.0001] (Figure 3D). Indeed, the tested high temperature resulted in a significant depression of leftward turning [within PI group comparison *t(364)* = *3.031*, adjusted *p* = 0.0078; red line] and increase in rightward turning [0.75 PI *t(364)* = *2.904*, adjusted *p* = 0.012; red line]. Conversely, low temperature depressed turn bias performance in the population (Figure 3D, blue lines). These results suggest a specific temperature-mediated change. However, in larval zebrafish, fluctuating temperature is a stressor, and elevation of stress signaling has been shown to attenuate visual bias in chickens (Rogers and Deng, 2005; Long et al., 2012; Haesemeyer et al., 2018). Therefore, we tested the effect of shaking on turn bias which is a potent stressor for larval zebrafish (Eto et al., 2014; Castillo-Ramírez et al., 2019; Apaydin et al., 2020). Sustained shaking during early development resulted in no population or turn bias magnitude changes (Figures 3E–G). Moreover, external temperature impacts the rate of zebrafish development, and based on previous studies, our conditions would lead to an estimated ±13 h shift in development (Kimmel et al., 1995). We show that our temperature assay results in a change in hatching, a developmental marker, yet no gross changes in morphology or survival (Supplementary Figures 3D–F). These data illustrate that etiologically relevant temperature fluctuations differentially and specifically affect inter-individual turn bias variation.

### Elevated Temperature Impacts Rostral Posterior Tuberculum Specification

**FIGURE 4:**
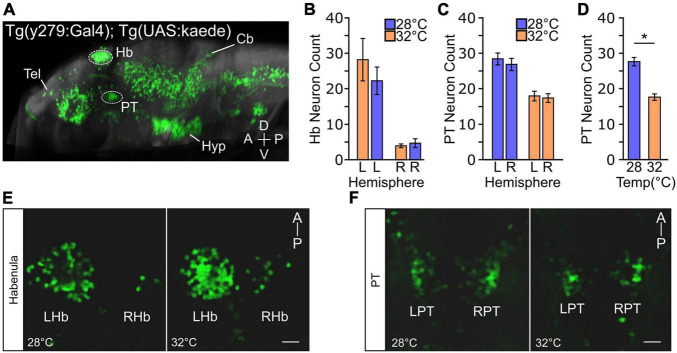
Temperature impacts y279 specified expression in the PT. **(A)** Single sagittal slice of larval zebrafish brain showing expression of enhancer trap *Tg(y279:Gal4)* obtained from the ‘Zebrafish Brain Browser’ atlas. Circled regions highlight the habenula (Hb) and rostral posterior tuberculum (PT) and white lines show the telencephalon (Tel), hypothalamus (Hyp), and cerebellum (Cb). **(B–F)** Effect of elevated temperature during early development on the expression of y279 in the habenula and PT. **(B)** Expression of y279 in the left and right hemisphere Hb nuclei (28°C purple, *N* = 16; 32°C orange, *N* = 14). **(C)** y279 positive PT neurons (28°C purple, *N* = 16; 32°C orange, *N* = 14). **(D)** Combined left and right hemisphere PT neuron counts (28°C purple, *N* = 16; 32°C orange, *N* = 14). **(E,F)** Representative images showing maximum intensity projections for y279 positive Hb (left habenula, LHb; right habenula, RHb) **(E)** and PT (left PT, LPT; right PT, RPT) **(F)** neurons for larvae raised at 28 or 32°C. Scale bar 20 μm. Asterisk *p* < 0.05.

A basic circuit involving the rostral posterior tuberculum (PT) and dorsal habenula (dHb) neurons has previously been described for zebrafish turn bias (Horstick et al., 2020). However, in wildtype larvae, no hemispheric differences in these neurons were found to account for left or right turning preference (Horstick et al., 2020). Because we found that elevated temperature disrupted left and right turning balance, we next wanted to determine if elevated temperature caused changes in neurons necessary for turn bias. We reasoned our environmental variables could alter neuronal development, as bias maintaining PT neurons are present as early as 2 dpf (Horstick et al., 2020), and dHb differentiation begins on 1 dpf (Gamse et al., 2003; Amo et al., 2010). First, we wanted to identify if a specific period during early development was sensitive to increased temperature. We found that elevated temperature during either 31–55 hpf or 55–79 hpf intervals did not recapitulate the population shift observed during the 1–4 dpf exposure (Supplementary Figure 4); therefore, we selected the full testing duration for further investigation. To visualize key dHb and PT neurons, we used the enhancer trap line *y279:Gal4*, which labels both populations of neurons (Horstick et al., 2020) (Figure 4A). In zebrafish, the left dHb is considerably larger than the right dHb (Gamse et al., 2003; Roussigné et al., 2009). We found that elevated temperature did not alter the habenula, and typical left/right asymmetry was observed [2-way ANOVA: interaction between temperature and hemisphere *F(1,56)* = *0.070*, *p* = 0.79; effect of hemisphere *F(1,56)* = *101.2*, *p* < 0.0001) (Figures 4B,E]. No hemispheric differences [main effect of hemisphere 2-way ANOVA *F(1,56)* = *0.493*, *p* = 0.49] were observed in the number of y279 positive PT neurons (Figure 4C). Therefore, we combined PT measures from both hemispheres. Interestingly, from these combined pools, y279 positive neurons in the PT were reduced after exposure to elevated etiological temperature during early development [high temperature 17.64 ± 0.885; normal temperature 27.59 ± 1.172: 2-tail *t*-test *t(58)* = *6.625*, *p* < 0.0001] (Figures 4D,F and Supplementary Figures 4C–F), establishing a potential neuronal basis for how high temperature during development modifies turn bias inter-individual variation.

### Motor Individuality Is Sensitive to Gene Signaling Associated With Neuronal Proliferation

Studies from *C. elegans* (Bertrand et al., 2011) and *Drosophila* (Linneweber et al., 2020) demonstrate that Notch signaling can generate functional asymmetries in the brain that drive unique individual behavioral responses. Established zebrafish mutant lines *mindbomb (mib)* and *mosaic eyes (moe)*, E3 ubiquitin ligase and Epb41l5 adapter, respectively, do not directly disrupt the Notch cascade, yet impair Notch signaling (Itoh et al., 2003; Ohata et al., 2011; Matsuda et al., 2016). Indeed, haploinsufficiency in these lines abrogates zebrafish turn bias, suggesting sensitivity to the levels of Notch signaling (Horstick et al., 2020). One of the canonical roles of Notch during early development is the regulation of neuronal proliferation (Appel et al., 2001; Mizutani et al., 2007; Yoon et al., 2008). Therefore, we next aimed to elucidate if turn bias is (1) sensitive to direct Notch antagonism in a dose-dependent manner and (2) if partial Notch inhibition impairs neuronal proliferation.

**FIGURE 5:**
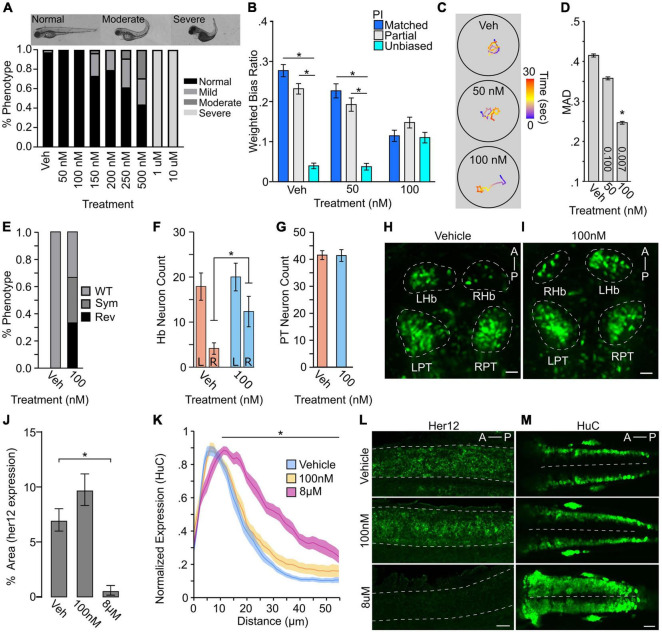
Turn bias is sensitive to levels of Notch signaling. **(A)** Phenotypic counts following Notch inhibitor treatment (Vehicle, *N* = 125; 50 nM, *N* = 88; 100 nM, *N* = 114; 150 nM, *N* = 123; 200 nM, *N* = 120; 250 nM, *N* = 101; 500 nM, *N* = 48; 1 μM, *N* = 43; 10 μM, *N* = 100). **(B)** Weighted absolute bias ratio averages (matched PI = 0,1 blue bar; Partial match PI = 0.25, 0.75 gray bar; unbiased PI = 0.50 cyan bar. **(C)** Illustrative traces for treatment groups. Scale bar color represents time in seconds. **(D)** Effect of Notch inhibition on MAD. *p*-values shown in bar, 1-way comparison of treatment groups to control. **(E)** Habenula symmetry for vehicle controls (*N* = 8) and 100 nM Notch inhibitor treated (*N* = 9) larvae showing proportion with WT, symmetric (Sym), or reversed (Rev) phenotypes. WT larvae have the larger habenula in the left hemisphere. y279 neuron counts in the habenula (**F:** vehicle *N* = 8; 100 nM *N* = 9) and PT (**G:** vehicle *N* = 8; 100 nM *N* = 9). For neuron counts, regardless of hemisphere the larger habenula was classified as the ‘left’ habenula. **(H,I)** Representative maximum projection images showing y279 labeled neurons in vehicle and control. Scale bar 20 μm. **(J)** Area of her12 expression in the spinal cord following LY411575 treatment (Vehicle, *N* = 12; 100 nM, *N* = 13; 8 μM, *N* = 11). **(K)** Normalized distribution of HuC/D positive neurons following notch inhibition (Vehicle: Blue, *N* = 18; 100 nM: Yellow, *N* = 13; 8 μM: Magenta, *N* = 13). *X*-axis distance spans half the spinal cord (0 micron = lateral spinal cord; 55 micron = spinal cord midline). Comparison shown is between vehicle and 8 μM along the whole length of black bar between matched positions. Ribbons ± SEM. **(L)** Representative images of her12 expression in 27 hpf embryos. Lateral view of spinal cord (dotted outline). Scale bar 20 μm. **(M)** Representative HuC labeling in 24 hpf embryos showing dorsal view. Dotted line denotes spinal cord midline. Scale Bar 40 μm. Asterisk *p* < 0.05.

To disrupt Notch signaling, we used the specific Υ-secretase inhibitor LY411575, which blocks the activation of the Notch signaling cascade (Geling et al., 2002; Fauq et al., 2007). Previous reports show that treatment with micromolar concentrations of LY411575 starting at mid-gastrulation results in a near-total loss of Notch signaling, which largely recapitulates the *mindbomb* mutant (Jacobs and Huang, 2019; Sharma et al., 2019). Therefore, we used 10 μM as a maximum dose and positive control for inhibitor efficacy across trials. To identify a level of Notch inhibition that could impair turning bias, we LY411575-treated larvae from mid-gastrulation to 4 dpf over 7 concentrations ranging from 50 nM to 10 μM and scored phenotypes at 3 dpf (Figure 5A). Developmental exposure of LY411575 up to 100 nM left most larvae morphologically normal, which we used as a maximum dose to test the impact on turn bias. Notch inhibition resulted in a significant change in TTA following the loss of illumination [1-way ANOVA *F(2,152)* = *4.614*, *p* = 0.011], causing an increase in overall turning at 100 nM inhibitor treatment compared to controls [vehicle 1175.95 ± 53.34, 100 nM 1411.39 ± 66.50: *t(152)* = *2.786*, adjusted *p* = 0.018] (Supplementary Figure 5). Whereas turn bias performance was reduced by Notch inhibition [main effect due to treatment 2-way ANOVA *F(2,144)* = *8.995*, *p* = 0.0002], with 100 nM inhibitor nullifying bias ratio strength differences due to PI, which was not observed at lower inhibitor concentrations (Figures 5B,C). In addition, 100 nM but not 50 nM treatment reduced overall inter-individual turn bias variation in the population (Figure 5D). This data suggests that a critical threshold of Notch signaling is required for generating variation in turn bias and overall performance, which is lower than levels necessary for normal gross morphological development. To identify a potential neuronal basis for the loss of bias following Notch inhibition, we LY411575-treated *y279:Gal4* embryos to quantify transgene positive dHb and rostral PT neurons, focusing on the inhibitor concentration that specifically impairs behavior yet not morphological development. Interestingly, we found that the levels of inhibition that abrogate bias also disrupts typical dHb hemispheric asymmetry, producing an increase in reversed and symmetric habenular phenotypes (Figure 5E). Similarly, we observed an increase in the smaller ‘right’ dHb neuron number [vehicle 4.25 ± 1.21, 100 nM 10.22 ± 1.64: *t(15)* = *2.870*, *p* = 0.012], consistent with increased habenular symmetry (Figures 5F,H,I). Conversely, the rostral PT was unaffected (Figures 5G–I).

To confirm that LY411575 exposure impaired Notch signaling, we examined *her12* expression, a downstream target of the Notch signaling cascade that is robustly expressed in the spinal cord, providing an unambiguous region to quantify expression changes (Jacobs and Huang, 2019). Exposure to micromolar inhibitor concentrations resulted in a near-total absence of *her12* expression, consistent with previous reports (Jacobs and Huang, 2019). The *her12* expression was, however, observed in the spinal cord of the 100 nM group at an intensity indistinguishable from controls (Figures 5E,L).

A canonical and conserved role for Notch during early development is regulating neuronal proliferation and maintaining progenitor pools, and the loss of Notch leads to increased proliferation (Appel et al., 2001; Cheng et al., 2004; Sharma et al., 2019). Therefore, we next wanted to determine whether the level of Notch inhibition that impairs turn bias individuality also disrupts proliferation. During zebrafish embryonic development, proliferative neurons are readily visualized in the anterior hindbrain using Elav (HuC/D) protein expression as a marker (Kim et al., 1996; Sharma et al., 2019). These proliferative neuron pools expand following high levels of Notch inhibition or in the *mindbomb* mutant background (Itoh et al., 2003; Sharma et al., 2019). Consistent to our observation with *her12* and PT neuron counts, partial pharmacological Notch inhibition (100 nM drug) induced no change in actively proliferating neurons, yet positive controls (8 μM) displayed robust expansion of proliferating neurons (Figures 5K,M).

**FIGURE 6:**
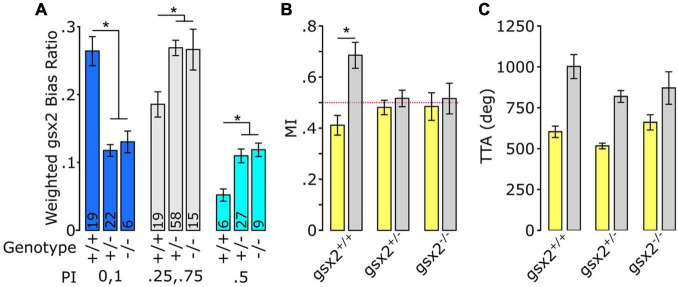
Loss of *gsx2* reduces inter-individual variation. **(A)** Effect of gsx2 genotype on weighted average bias ratio per performance groups (Matched: PI = 0,1; Partial PI = 0.25,0.75; and Unbiased PI = 0.5). Numbers on bars indicate *N*. Weighing was based on the percent of larvae within each PI per genotype. **(B)** MI shows that only gsx2+/+larvae maintain persistent same-direction turning following the loss of illumination. Dotted line at 0.5 indicates random movement. **(C)** TTA between genotypes is not affected (gsx2+/+, *N* = 45; gsx2±, *N* = 113; gsx2–/–, *N* = 32). Baseline (yellow) and dark (gray) responses in **(B,C)**. Asterisk *p* < 0.05.

Notch signaling is ubiquitous in the larval zebrafish nervous system (Tallafuss et al., 2009; Banote et al., 2016; Kumar et al., 2017), and pharmacological inhibition is not specific. Consequentially, we next aimed to determine whether proliferative pathways in restricted areas of the brain may also contribute to turn bias. Genomic screen homeobox transcription factors (Gsx1 and 2, formerly Gsh1 and 2) are affecters of the Notch signaling pathway in mouse, and Gsx2 maintains neural progenitor pools in the developing telencephalon (Wang et al., 2009; Pei et al., 2011; Roychoudhury et al., 2020). In larval zebrafish, *gsx2* is predominantly expressed in the pallium, preoptic area, hypothalamus, and hindbrain, with an established putative null TALEN deletion mutant line (Coltogirone et al., 2021). As *gsx2* mutants show no gross morphological abnormalities during larval stages, we used these lines to test turn bias. Heterozygous and mutant *gsx2* larvae displayed reduced inter-individual variation and a shift toward less persistent turn bias (Figure 6A). The loss of persistent same-direction turning was similarly observed using match index (MI), an analogous metric (Figure 6B). Yet, TTA during light-search was not significantly changed across genotypes [1-way ANOVA *F(2,187)* = *2.730*, *p* = 0.068], suggesting the loss of same-direction turning is not due to reduced light-driven behavior (Figure 6C). Thus, our analysis implies that broad and local haploinsufficient changes in Notch signaling and Gsx2 contribute to inter-individual variation in turn bias behavior, independent of canonical roles in proliferation.

## Discussion

Here we reveal that during light-search initiated by the loss of illumination, larval zebrafish exhibit significant inter-individual variation in turn bias, a handed-like behavior. Based on our newly developed assays, we were further able to show mild changes in search behavior correlated with left and right turning types. However, the impact of turning on search motor patterns was specific, as we found no evidence of individual motor changes during baseline illumination, consistent with previous studies (Horstick et al., 2020). We demonstrated a turn bias spectrum across the population which shows the previously described left/right turning types (Horstick et al., 2020). In addition, our analysis revealed a consistently unbiased turning type, supported by multiple independent recording strategies (4×, 8×, and q4×). Furthermore, we show that temperature changes during early development result in sustained changes in inter-individual variation. Finally, we tested how signaling pathways associated with neuronal proliferation affected turn bias development, using either pharmacological inhibition of Notch signaling or a presumable null Gsx2 mutant. Notch and Gsx2 represent canonical broad and regional regulators of proliferation, respectively. Interestingly, turn bias attenuation is observed with partial Notch inhibition and in *gsx2* heterozygotes, suggesting dose-dependent sensitivity. Despite a well-established role for Notch in cell proliferation, the inhibitor concentrations that selectively impairs turn bias did not result in observable changes in proliferation, at least early in development (see Figure 5). Our findings confirm that three turning types can be categorically defined, are modulated by specific etiological relevant environmental cues, and are sensitive to internal proliferative associated signaling pathways. One potential caveat is that zebrafish strains are not isogenic and maintain some genetic heterogeneity (Butler et al., 2015), potentially contributing to inter-individual differences. Nevertheless, our work develops larval zebrafish as a powerful model to identify mechanisms generating inter-individual variation in vertebrates.

### Determination of Bias

**FIGURE 7:**
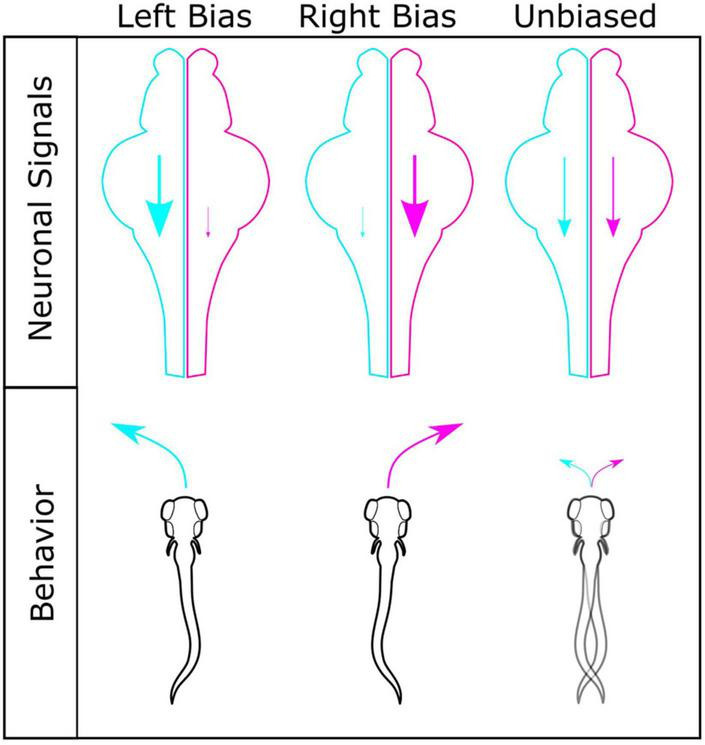
Model for generating different turning types. Interhemispheric differences in turn bias driving motor signals are a potential mechanism for establishing turning types. Left (cyan) and right (magenta) hemispheres shown for left, right, and unbiased motor types, with corresponding motor drive shown by scale of descending arrow. For individuals with robust left or right turning types, a strong ipsilateral turn bias signal persists in a single hemisphere, with limited conflicting input from the contralateral hemisphere. In individuals with similar turn bias drive from both hemispheres, no single turn direction persists due to conflict between hemispheres, resulting in lower bias ratios and unbiased turning types.

Our findings suggest a ‘hemispheric noise’ model where turn bias and inter-individual variation is modulated by conflicting brain hemisphere signals in turn bias driving neurons (Figure 7). We elucidated that change in bias ratios strength distinguishes unbiased versus biased larvae. Moreover, we establish that this change is not a result of a loss of photo-responsiveness in unbiased individuals (total turning, see Supplementary Figure 1); rather a failure to navigate in a single direction during light-search consistently. This observation supports the conclusion that unbiased individuals are not a subset with impaired photo-responsiveness, but a distinct behavioral motor profile during search behavior. Supporting this model, when we quantify the strength of individual trials, the bias ratios exhibit a step-wise decrease, i.e., PI 1 < 0.75 < 0.5, suggesting accumulating inter-hemispheric noise that degrades overall individual bias persistence. Corroborating this model, previous studies showing that unilateral ablation of rostral PT neurons, which are required for turn bias in larval zebrafish, increases turning strength in the direction ipsilateral to the intact neurons, indicating ablation removes conflicting input from the contralateral hemisphere (Horstick et al., 2020). In pigeons, a classic model for hemispheric specialization and individual variation (Güntürkün et al., 1998; Freund et al., 2016), increased conflict between hemispheres exacerbates visual task latency (Manns and Römling, 2012). Therefore, variable balance in hemispheric signaling may be a conserved mechanism in generating inter-individual variation (Chen-Bee and Frostig, 1996; Linneweber et al., 2020). Inter-hemispheric communication is vital for the function of the visual system (Bui Quoc et al., 2012; Chaumillon et al., 2018), including photo-driven behavior in larval zebrafish (Gebhardt et al., 2019). The counter hypothesis is a ‘switching model’ where unbiased larvae would display vigorous directional turning, yet in randomly selected directions over sequential trials. This model is consistent with a ‘winner take all’ circuit function (Fernandes et al., 2021). Indeed, within the primary visual processing center in zebrafish, the optic tectum, neurons operate in a winner take all style during visually guided behavior (Fernandes et al., 2021). However, turn bias is driven by the loss of visual cues that activate rostral PT neurons, which do not project to the tectum (Horstick et al., 2020), implicating that even though turn bias is visually evoked, the mechanism is likely independent of a tectal winner take all mechanism. Despite the neurons maintaining zebrafish turn bias being identified, the underlying mechanism imposing a specific turning type remains unknown (Horstick et al., 2020). Our analysis suggests a model of competitive inter-hemispheric communication modulating the magnitude of inter-individual turn bias variation that is further adjusted by fluctuating and specific variables in the internal and external environment.

### Regulation of Individuality

The mechanisms driving unique individual behavioral responses based on sex or sensory context are well described (Asahina et al., 2014; Lewis et al., 2015; Yapici et al., 2016; Marquart et al., 2019; Ishii et al., 2020; Nelson et al., 2020). However, why individuals in a population will show variable response types to a consistent stimulus is poorly understood, especially in vertebrates. One of our goals was to determine what internal and external elements may modulate turn bias variation as a basis to explain how different response types potentially arise. To test external environmental factors, we selected environmental enrichment, social experience, temperature, and salinity. One hypothesis for how factors like enrichment or social experience influence inter-individual variation is through micro-environmental interactions that create unique individual experiences (Kain et al., 2015). However, our data indicate that these interactions do not influence turn bias variation in zebrafish. One possible explanation is that during early development, 1–3 days post-fertilization, larvae are primarily inactive and only begin actively exploring around 4 days post fertilization (Colwill and Creton, 2011; Lambert et al., 2012). Conversely, responsiveness to conspecifics is not observed until 3 weeks (Dreosti et al., 2015; Larsch and Baier, 2018). As bias is established by 4 dpf, the underlying mechanisms may no longer be malleable to environmental experiences beyond this developmental interval.

We also tested temperature and salinity, emphasizing etiological ranges that zebrafish could experience in their native environments (Engeszer et al., 2007; Sundin et al., 2019). Salinity and temperature are critical environmental determinants and have been shown to drive evolutionary changes in stickleback populations (Gibbons et al., 2017). However, we found that only raising larvae at varying temperatures resulted in modifications to inter-individual variation. We show that temperature-dependent effects are not a generic thermal stress response. Etiological increases in temperature have been shown to attenuate turn bias in adult reef fish, implicating a potentially broader thermal sensitivity in bias establishing mechanisms (Domenici et al., 2014). Our analysis establishes that early developmental exposure to etiological temperature fluctuation results in sustained and specific turn bias changes.

Intriguingly, the specification of habenular hemispheric asymmetry is sensitive to the rate of development (Aizawa et al., 2007), and developmental rate is temperature sensitive (Kimmel et al., 1995). This observation could provide a potential mechanism for thermal driven changes in turn bias. However, our testing conditions produced no gross change in the habenula nuclei morphology. This observation, however, does not exclude functional or subcellular changes. Surprisingly, we observe a bilateral reduction in *Tg(y279)* positive PT neurons, which are essential for maintaining turn bias, in the elevated temperature experiments. A primary function of the PT is to integrate diverse sensory inputs (Striedter, 1991; Derjean et al., 2010; Yaeger et al., 2014). However, thermosensitivity of the PT neurons has not been previously described, and we believe this is a novel observation. Future studies identifying the genetic basis of the *Tg(y279)* enhancer trap, which is currently unknown, will be instrumental in elucidating how temperature impacts PT neuron specification and inter-individual variation. The specific abrogation of leftward turning types in increased temperature conditions provides a powerful model to interrogate underlying neural changes in a vertebrate brain associated with individual behavioral patterns.

Last, we wanted to identify molecular signaling pathways regulating turn bias. Biased turning in larvae is largely lost in heterozygotes of mutant lines associated with Notch signaling, yet the impact of direct Notch inhibition was unexplored (Horstick et al., 2020). In *Drosophila* and *C. elegans*, Notch signaling is essential for establishing individual visual navigational strategies and asymmetric chemosensory neuron identities, respectively (Bertrand et al., 2011; Linneweber et al., 2020). Thus, work from several species implicates Notch as a driver of variation at behavioral and neuronal levels. Indeed, we show that partial Notch disruption, using a specific pharmacological inhibitor, disrupts biased turning in larval zebrafish, yet not the ability to respond to illumination changes, establishing a direct role of Notch signaling for turn bias, which is independent of gross morphological development. Despite the established role of Notch in neural proliferation, we found no significant change in proliferative neurons, *her12* expression, or number of PT neurons at the dosages used for behavioral studies. However, we did observe disruption to the typical left/right hemispheric asymmetry of the habenula, observing an increase in reversed or symmetric habenula. Interestingly, similar disruption to habenula symmetry is observed in *mib* zebrafish which have severely reduced Notch signaling (Aizawa et al., 2007). Our results, show a novel Notch dose-sensitivity for habenula asymmetry development, which may be a potential neural basis for the absence of biased behavior following low levels of Notch inhibition. Since Notch signaling is essential for diverse cellular functions, and the precise downstream signaling mechanisms are highly sensitive to the strength of Notch signaling (De Smedt et al., 2005; Shen et al., 2021), the low inhibitor concentrations used may be sub-threshold for disturbing the spatial-temporal patterns of *her12* and HuC/D tested here. In addition, the downstream effects of Notch are dependent on the cellular micro-environments, determined by the local co-expression of Notch receptors, ligands, and auxiliary proteins (Demehri et al., 2009; Bertrand et al., 2011; LaFoya et al., 2016). Therefore, the levels of Notch reduction that impair turn bias, but not morphology, may not be sufficient to alter Notch associated mechanisms impacting proliferation. Nevertheless, subtle changes in Notch could lead to changes in cellular micro-environments, thereby altering downstream signaling cascades, and ultimately impacting turn bias maintaining neurons. Notch haploinsufficiency is known to generate a myriad of defects and disease states, including vasculature defects, seizure, autism, and brain malformations, demonstrating that reduced Notch signaling can disrupt biological functions (Krebs et al., 2004; Connor et al., 2016; Fischer-Zirnsak et al., 2019; Blackwood et al., 2020). However, the pharmacological inhibition used in our current study is not regionally specific. Therefore, we also tested an established zebrafish *gsx2* mutant line, and *gsx2* is predominately expressed in subsets of hypothalamic, preoptic area, pallium, and hindbrain neurons (Coltogirone et al., 2021). The reduction in turn bias in *gsx2* heterozygotes and mutants suggests that turn bias variation is sensitive to local changes in brain regions where *gsx2* is expressed, independent of the previously described rostral PT and habenula (Horstick et al., 2020). As the levels of Notch that reduce turn bias do not impact proliferation, it seems possible that Notch and Gsx2 modulate turn bias by independent mechanisms. Our current analysis identifies two conserved molecular signaling and transcriptional control mechanisms, Notch and Gsx2, and novel neuroanatomical substrates as important for generating variation in turn bias.

### Function of Turn Bias and Inter-Individual Variation

Behavioral variation is observed in diverse species and behavioral modalities (Byrne et al., 2004; Elnitsky and Claussen, 2006; Cauchard et al., 2013; Horváth et al., 2020). In zebrafish, even complex neuromodulatory processes such as startle habituation display inter-individual variation with distinguishable ‘habituation types’ (Pantoja et al., 2016, 2020). Yet, the general question remains, “why do specific behavioral modalities manifest inter-individual differences?” Considering a simple form of inter-individual variation, such as turn bias, may offer insights to these questions. Zebrafish are active hunters during larval stages and predatory success depends on visual input, thus establishing a potent drive to remain in illuminated areas (Gahtan et al., 2005; Filosa et al., 2016; Muto et al., 2017). Following the loss of light and of overt navigation cues, larvae initiate a local light-search, where individual turn bias is triggered, causing looping trajectories (Horstick et al., 2017). Looping search trajectories are observed in various species in the absence of clear navigational cues, suggesting an efficient systematic strategy (Collins et al., 1994; Conradt et al., 2000; Zadicario et al., 2005). However, even seemingly optimal behaviors may not be advantageous in all contexts (Simons, 2011). Variation in turning types may ensure individuals across the population possess strategies to mitigate erratic environmental challenges, a form of bet-hedging (Simons, 2011; Kain et al., 2015). Similarly, behavioral variation adds unpredictability to a population. Predictable behavioral patterns can be exploited by predators (Catania, 2009, 2010). For aquatic species, this may be advantageous as some heron species, a predator of small fish, use a canopy hunting strategy, covering the water surface with their wings and blocking light (Kushlan, 1976). Prey populations with unpredictable responses would potentially provide a more challenging target (Humphries and Driver, 1970). Even though larval fish may not be the intended target of heron canopy hunting, larval behavioral patterns may persist over their lifespan. Indeed, adult zebrafish display a persistent turn direction preference (Fontana et al., 2019), although the correlation to larval turn bias is currently unexplored. Ultimately, the etiological purpose for turn bias variation is most likely a combination of multiple explanations, including bet-hedging, generating unpredictability, and genetically encoded sources of variation.

## Data Availability Statement

Inquiries can be directed to the corresponding author for R scripts and datasets generated in this study.

## Ethics Statement

The animal study was reviewed and approved by West Virginia University Institutional Animal Care and Use Committee.

## Author Contributions

EH conceived the experiments. EH and JH wrote the manuscript and analyzed data. SL wrote custom R scripts for data analysis. SB provided *gsx2* lines and associated analysis. JH, MW, JS, HC, HP, LB, and CS performed experiments. All authors contributed to the article and approved the submitted version.

## Conflict of Interest

The authors declare that the research was conducted in the absence of any commercial or financial relationships that could be construed as a potential conflict of interest.

## Publisher’s Note

All claims expressed in this article are solely those of the authors and do not necessarily represent those of their affiliated organizations, or those of the publisher, the editors and the reviewers. Any product that may be evaluated in this article, or claim that may be made by its manufacturer, is not guaranteed or endorsed by the publisher.
